# A multimodal driver monitoring benchmark dataset for driver modeling in assisted driving automation

**DOI:** 10.1038/s41597-024-03137-y

**Published:** 2024-03-30

**Authors:** Khazar Dargahi Nobari, Torsten Bertram

**Affiliations:** TU Dortmund University, Institute of Control Theory and Systems Engineering, Otto-Hahn-Str. 8, 44227 Dortmund, Germany

**Keywords:** Interdisciplinary studies, Human behaviour, Databases, Electrical and electronic engineering, Scientific data

## Abstract

In driver monitoring various data types are collected from drivers and used for interpreting, modeling, and predicting driver behavior, and designing interactions. Aim of this contribution is to introduce *manD 1.0*, a multimodal dataset that can be used as a benchmark for driver monitoring in the context of automated driving. manD is the short form of human dimension in automated driving. *manD 1.0* refers to a dataset that contains data from multiple driver monitoring sensors collected from 50 participants, gender-balanced, aged between 21 to 65 years. They drove through five different driving scenarios in a static driving simulator under controlled laboratory conditions. The automation level (SAE International, Standard J3016) ranged from SAE L0 (no automation, manual) to SAE L3 (conditional automation, temporal). To capture data reflecting various mental and physical states of the subjects, the scenarios encompassed a range of distinct driving events and conditions. *manD 1.0* includes environmental data such as traffic and weather conditions, vehicle data like the SAE level and driving parameters, and driver state that covers physiology, body movements, activities, gaze, and facial information, all synchronized. This dataset supports applications like data-driven modeling, prediction of driver reactions, crafting of interaction strategies, and research into motion sickness.

## Background & Summary

The first problem with machine analysis of human state is the collection of data that encompasses the entire scene^1^. Therefore, a robust dataset about the driver is crucial for an accurate estimation of driver state. Such a dataset should be sufficiently large to yield statistically significant conclusions, be comprehensive by encompassing all pertinent information about the driver and the driving environment, maintain accuracy in its details, and be highly reliable.

For several decades, literature has addressed the concept of driver monitoring. Initially, the primary objective of driver monitoring was to study human driving behavior in manual driving modes across various situations. As a result, datasets gathered for this purpose mainly encompassed vehicle-related information, such as the status of the pedals, steering wheel, and overall vehicle dynamics^2^. However, as the focus evolved, monitoring the driver state within the vehicle gained significance. This shift recognized that driver state factors, such as fatigue, could profoundly influence behavior during manual driving^3–5^. Consequently, datasets began to incorporate factors like attention and distraction, which hold relevance in both manual and automated driving contexts. While some datasets include merely RGB (color space, combination of red, green, and blue colors) images with corresponding labels^6–9^, others offer a more comprehensive view, integrating IR (infrared) or depth images alongside RGB images^10^. These visuals facilitate the extraction of insights like gaze direction, drowsiness indicators, hand-wheel interactions, and other contextual data, all of which help in assessing driver attention. A significant distraction source while driving is preoccupation with a non-driving-related task (NDRT), leading some datasets to categorize driver activities during both manual and automated driving phases^11–14^. Beyond camera footage, physiological data from the driver can furnish further insights, particularly about their cognitive and emotional states^15–19^. It’s essential to consider not just data about the driver but also about the vehicle, its driving dynamics, and environment to capture a holistic understanding of both the driver and the driving situation^20^.

Table 1 gives an overview of the characteristics of the frequently cited datasets for driver monitoring and the introduced dataset. Most datasets have notably been created to identify specific, limited factors, such as drowsiness. However, various driver state factors, including attention, workload, and emotion, can interplay and influence driving behavior. These should be assessed collectively. A comprehensive dataset should encapsulate multiple driver state factors. Additionally, elements like driving dynamics and environmental shifts, including traffic and weather conditions, can influence the driver state and should be mentioned in the dataset synchronized with other data points. These elements are invaluable for a deeper understanding and interpretation of the driver’s behavior. Hence, it’s not only beneficial but sometimes essential to incorporate these elements into the monitoring dataset. Furthermore, data should be obtained from a diverse sample, encompassing a broad range of driving scenarios and driver states, ensuring subsequent analyses and findings are holistic and representative. While creating the manD 1.0 dataset, all of the mentioned aspects are taken into account to obtain a comprehensive dataset for driver monitoring.

**Table 1:** Overview of the characteristics of the frequently cited datasets for driver monitoring compared to manD 1.0.

Dataset	Size	Objective measures	Subjective measures	Available labels	Further included data
FDUDrivers^8^	100	RGB image	—	—	Environment, vehicle
Vehicle driving behavior^2^	—	Acceleration, angular velocity	—	Driving intention	—
Driver behavior dataset^11^	—	RGB image	—	5 activities	—
Howdrive 3D^6^	9	RGB image	—	10 activities	—
Distracted driver dataset^9^	44	RGB image	—	10 activities	—
State farm distracted driver detection^7^	—	RGB image	—	10 activities	—
DAD^46^	31	Depth, IR images	—	Anomalous action	—
DMD^10^	37	RGB, depth, IR images	—	13 activities	—
3MDAD^13^	50	RGB, IR iimages	—	16 activities	—
Drive&Act^14^	15	RGB, depth, IR videos, 3D body pose	—	83 activities	—
NITYMED^3^	21	Video	—	Yawning, microsleep	—
YAWDD^4^	107	Video	—	Yawning	—
Driver drowsiness dataset^5^	28	RGB image	—	Drowsiness	—
DriverMVT^18^	9	HR, video	—	Drowsiness	Vehicle
Driving fatigue dataset^47^	20	RGB image, HR	3-point fatigue self-report	Fatigue	—
DriveAHead^12^	20	Depth, IR images, head pose	—	—	—
MDM^20^	59	Video, head pose, gaze behavior, RGB, depth images	—	—	Environment, vehicle
Physiological and emotional states^15^	10	HR, EDA	5-point stress self-report	Stress level	Vehicle
Warwick-JLR^17^	20	ECG,EDA		Workload	Vehicle
Detecting stress during real-world driving^16^	24	Respiration, ECG, EMG, EDA	5-point & 7-point stress self-report	Driving stress	—
DEFE^48^	60	Video	DES, SAM^49^	Emotion	—
A Multimodal Dataset for Various Forms of Distracted Driving^19^	68	Thermal, RGB images, EDA, respiration, HR, gaze behavior	—	Distraction, emotion	Vehicle
manD 1.0^26^	50	EDA, PPG, ECG, EEG, seat pressure, RGB image, gaze behavior	DES	Activity, emotional effect, environmental event	Environment, vehicle

In the literature, a large number of varying factors are attributed to the driver state, depending on the research focus or data availability. However, many of these factors either overlap with one another or are context-dependent, rendering them not universally representative and well structured. In determining the most pertinent factors to depict the driver state for this dataset, we took into account established driver models and cognitive architectures. Adaptive Control of Thought-Rational (ACT-R)^21^, Queueing Network-Model Human Processor (QN-MHP)^22^, and CLARION^23^ are a few models recurrently employed for both qualitative and computational research^24^. ACT-R is a cognitive architecture that designates multiple modules to the driver, encompassing the goal, memory, perceptual, and motor functions. Conversely, QN-MHP offers a framework for the mathematical modeling of the driver, defining an array of interconnected queues that denote varied cognitive levels. These queues are categorized as perceptual, motor, or central, with the central queue enveloping cognitive processes like attention, memory, and decision-making. CLARION, on the other hand, outlines a cognitive architecture where modules for perception, action, motor functions, emotion, and goal synergistically interact to shape driver behavior. Considering insights from all three models results in a driver model that underpins the selection of pertinent driver state factors^25^. Human information processing takes place in three stages: sensory perception, decision-making, and motor response. The factors that determine the driver state are visual attention for sensory perception, emotion, attention, and workload for decision-making, and the driver’s activity and the position of body parts for motor response^25^. Table 2 offers a comprehensive overview of these driver state factors, the associated measuring sensors, and potential scaling features.

**Table 2:** Overview of driver state factors, which are extracted from the driver’s cognitive architecture and mental functions as the most pertinent factors; sensors, which are mainly employed in the literature to measure the corresponding driver state factor; and scaling features, which are the computed features extracted from sensor data.

Driver state factors		Sensors	Features
Sensory	Visual	Eye tracker	Gaze direction
Decision-making	Emotion	Camera Physiological sensors	*HR*^50,51^
			*HRV*^52^
			*SCL*^50,51^
			Skin temperature^53^
			*EEG*^54^
	Attention	Eye tracker Physiological sensors	*EEG*^55^
			Blink rate^56^
			Horizontal gaze deviation^57^
			Eye-off-road time^58^
	Workload	Eye tracker Physiological sensors	Pupillary^59^
			*HR*^59^
			Skin resistance^59^
			Nose temperature^60^
			Blink latency^61^
			PERCLOS^61^
			Fixation duration^61^
			Blink duration and rate^61^
			Horizontal gaze dispersion^62^
			Percent road center (PRC)^62^
			Distribution of eye fixation^63^
Motor	Position Activity	Camera Seat-pressure-sensors	Pressure distribution^64,65^

**Fig. 1:**
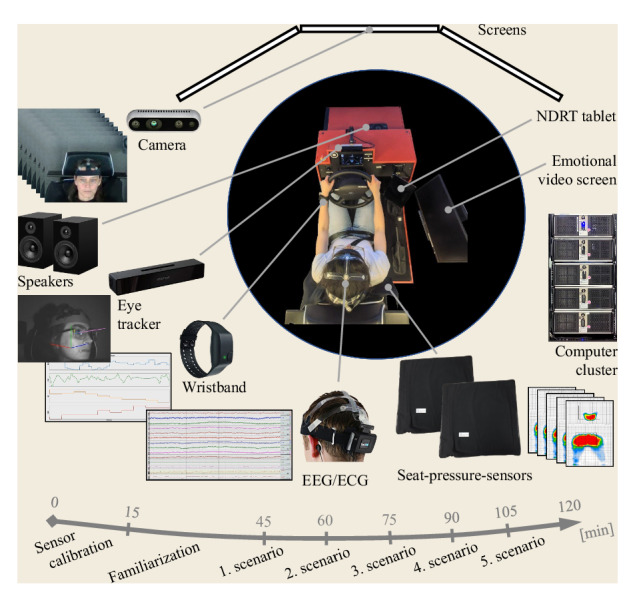
Schema of the study and the employed sensors.

Fig. 1 shows a schema of the study and the employed sensors. The experiment was conducted using a static driving simulator in the laboratory under controlled conditions. Out of the 50 subjects who participated in the study, 11 experienced motion sickness, and their data has been reported separately in the dataset. The other participants drove through five predefined scenarios. Data related to the driver, the (simulated) vehicle, and the environment were recorded throughout the driving sessions and have been included in the presented dataset after appropriate preprocessing.

manD 1.0^26^ can support a variety of research topics concerning human drivers. These include modeling driver state factors and driving behavior, predicting reaction time and type, and exploring the correlations and interrelationships among different driver state factors, for drivers with or without motion sickness.

## Methods

### Experimental setup

The experiment is conducted in a driving simulator under controlled laboratory conditions at a constant temperature of 22. The driver’s cabin is separated from the rest of the environment to simulate the vehicle cabin, and distractions and disturbances have been kept to the minimum. The ambient light is turned off, but in the simulator a light is available to the driver that mimics the light source in the vehicle. The equipment used is explained below.

#### Driving simulator

The static driving simulator consists of a driving mockup with three 55″ displays. The displays are situated in front of the driver’s seat, each angled at 120 from one another, providing the driver perspective. The entire mockup is separated from its surroundings to reinforce the sense of presence. Automatic mode during manual driving is facilitated, obviating the need for gear and clutch engagement. During the simulation, in addition to manual driving (SAE L0), automated driving in SAE L1 to L3 is offered as well. Beside the driving equipment, an extra pedal is attached to the right of the gas pedal that serves as a communication interface to the automated system. With this pedal, drivers can robustly answer the automated system’s yes-no questions. A tablet is placed to the right of the steering wheel, providing a gaming interface as NDRT. Additionally, an extra display is positioned on the driver’s right side, playing videos to invoke specific emotional states in drivers before they embark on their ride, thus contributing to the psychological aspects of the simulation. Furthermore, a book, a bottle of water, and cookies are placed within easy reach of the drivers.

The simulator employs the SCANeR studio 2021 software (AVSimulation, Boulogne-Billancourt, France) as a real-time simulation platform. SCANeR’s connectivity with external sensors is enabled by application programming interfaces (APIs) developed using the Python programming language.

#### Intel RealSense: camera

An Intel RealSense D435 camera (Intel Corporation, Santa Clara, California, U.S.) is mounted on top of the front display 1.5 away from the driver to capture the facial behavior of the participants. RGB images are captured by the camera at a frame rate of 30 FPS and a resolution of 1920 × 1080 px.

#### SmartEye: eye tracking system

A SmartEye Aurora eye tracking system (SmartEye AB, Gothenburg, Sweden) is fixed on the driving mockup on top of the dashboard directly in front of the driver at a distance of about 0.7 from driver’s eyes. The system utilizes the dark pupil and corneal reflection as an eye tracking principle and has a sampling rate of 120. A SmartEye Pro 9.3 software is supplied with the eye tracker system, which receives data from the eye tracker via a cable and communicates the data with the SCANeR software in real time.

#### Empatica E4: photoplethysmogram/electrodermal activity sensor/3-axis accelerometer

In this study, a wearable Empatica E4 (Empatica Inc., Boston, Massachusetts, U.S.) wristband is applied to collect physiological data from drivers. The wristband is equipped with a photoplethysmogram (PPG) sensor to measure blood volume pulse (*BVP*), from which the interbeat interval (*IBI*), heart rate (*HR*), and heart rate variability (*HRV*) can be derived. In addition, the constantly fluctuating changes in the electrical properties of the skin can be monitored via an electrodermal activity (*EDA*) sensor placed in the wristband. *EDA* value depends on material of electrodes, position of the sensor, and environmental conditions such as temperature and humidity. Furthermore, the integrated 3-axis accelerometer and infrared thermopile record participants’ arm movement and peripheral skin temperature. The collected data from the Empatica E4 are transmitted via Bluetooth v.4.0 to the E4 Streaming Server software and from there to the SCANeR software via a Python API in real time.

#### BIOPAC: electroencephalogram/electrocardiogram

**Fig. 2:**
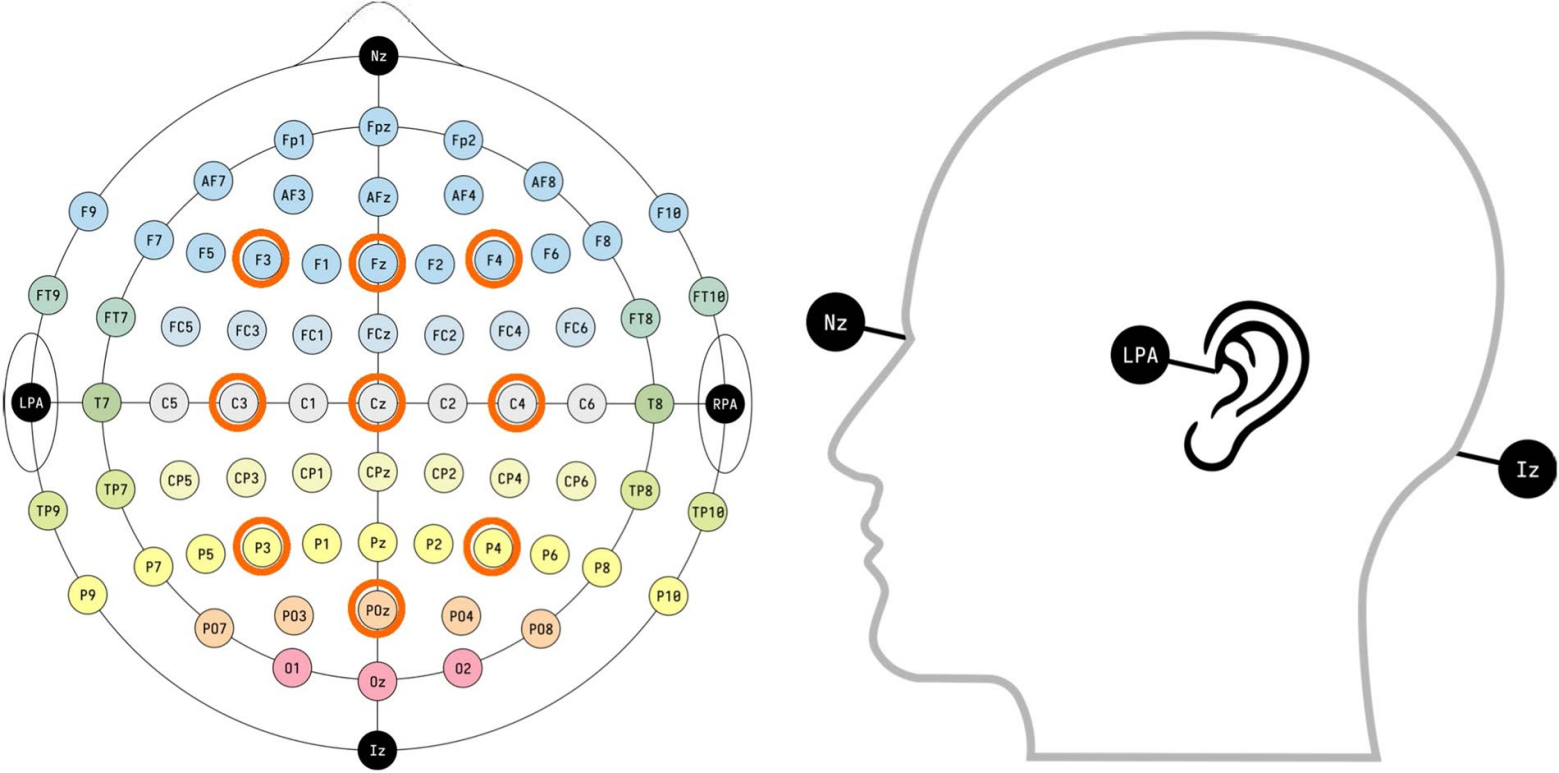
Distribution of the *EEG* electrodes based on International 10-10 system^66^.

Participants are also asked to wear a B-ALERT X10 wireless electroencephalogram (*EEG*) sensor (BIOPAC, Goleta, California, U.S.) to record their brain activity during the experiment. The sensor has nine wet *EEG* channels (*Poz, Fz, Cz, C3, C4, F3, F4, P3, P4*) and an electrocardiogram (*ECG*) channel to capture heart activity as well. Fig. 2 illustrates the distribution of the *EEG* electrodes color coded according to the lobes of the brain. Two mastoid electrodes are placed behind the ears. The sensor delivers measurements via Bluetooth to AcqKnowledge 4 software and subsequently to the SCANeR in real time.

#### BodiTrak: seat-pressure-sensor mats

Two BodiTrak2 Pro seat-pressure-sensor mats (Vista Medical Ltd, Winnipeg, Manitoba, Canada) are integrated into the acquisition system: one is placed on the driver’s seat and the other on the driver’s backrest. Each mat comprises 1,024 pressure sensors (arranged in a 32 × 32 grid) and spans an area of 0.45 × 0.45. For optimal performance and precision, the seat mat is calibrated to 200, while the backrest mat is set at 100. With each calibration, accuracy at the midpoint of the calibration range is maintained at 10% of the calibration maximum. Thus, for example, for the backrest mat, the highest measurable pressure stands at 100, and the accuracy at the 50 level is 10. Pressure readings are transmitted to a computer via a USB 2.0 connection, with a frequency reaching up to 150, and then forwarded to SCANeR through a Python API in real time.

### Experimental procedure

The study is first approved by the ethics commission of TU Dortmund University. An informed consent is also obtained from the participants before start of the experiment. The prerequisite for participation in the experiment is basic English knowledge and the possession of a driver’s license. A couple of the participants got motion sickness during the experiment, especially right at the beginning of the experiment. Thus, they were asked to terminate the experiment. The data collected from this group are provided separately in the dataset for the case of motion sickness research.

**Fig. 3:**
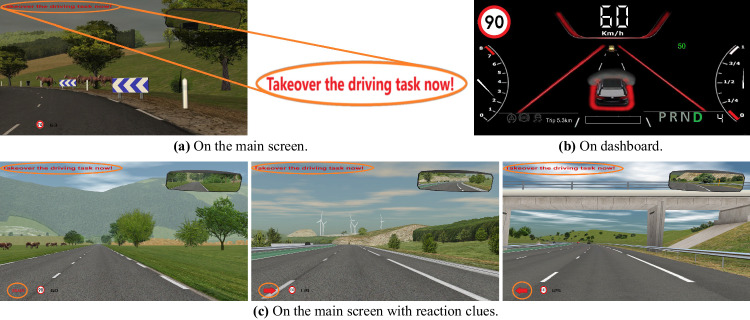
Visual modalities of TOR.

The study lasts about two hours for each participation. As first part of the experiment, participants are adequately informed about the goals of the experiment, the procedure, and the data collection by the sensors and the signed consent form is collected. The sensors are then connected and calibrated for each driver. Next, the drivers have the opportunity to drive in the simulator, familiarize themselves with the driving and automated functions, and get used to driving in the virtual environment. Subjects drive once manually with automatic setting and then in automated mode from SAE L1 to SAE L3. They are free to drive as many times as they want until they feel comfortable with driving in the simulator. During the familiarization drive, participants experience takeover request (TOR) and familiarize themselves with the available NDRTs. The TOR has, in addition to the acoustic modality composed of warning beeps and speech, also visual modality in the form of text on the main screen and color effects on the dashboard (see Fig. 3a,b). Three activities are selected as NDRT: the auditory digit-span task^27^ requested by the automated system during the experiment, the n-back game^28^, and the Subway Surfers game (co-developed by Kiloo and SYBO Games, released 2012), which are available on the fixed tablet for the driver at any time and reminded by the automated system only on predefined occasions. Drivers are asked to drive realistic and consider the experiment as a real driving situation. Afterwards, they drive five predefined driving scenarios one after the other in alternating order with breaks in between. Before and after scenarios participants fill Differential Emotions Scale (DES)^29^ questionnaire to subjectively assess their emotions and they are instructed to rate their current feelings. They are also asked verbally by automated system about their emotions during the scenarios to ensure that certain previously evoked emotions are faded. Before some of the scenarios, a video clip is played to the drivers to evoke emotions in the drivers. During the driving scenarios, subjects receive instructions or cues from the automated system about the driving situation and available features, but drivers are free to accept the instructions and choose their preferred response and activity.

### Design of experiment

This study aims to create a statistically reliable, multimodal dataset from drivers. A synchronized multi-sensor system is utilized to monitor the drivers, capturing an expansive set of driver state factors across diverse driving situations. This comprehensive approach guarantees a profound understanding of the driver, vehicle, and environment states. Besides, the design’s meticulous structure enhances the statistical reliability of the acquired data.

Participants are briefed on the study’s general objectives and procedures. However, the details and focus points are not disclosed until after the experiment concludes, to maximize the validity of the gathered data.

The research design incorporates various factors, including driver, vehicle, and environment states’ factors. These factors manifest in several types, spanning both qualitative (ordinal/nominal) and quantitative (continuous/discrete) variables. Covariates and potential confounding variables are accounted for as much as possible within the experiment. Age and gender, as two characteristics of the participants are considered covariates and are addressed via stratification^30^. Other characteristics, such as height and body mass index (BMI), are reported in the dataset but not considered as covariates, as these variables were unknown before the study. Laboratory conditions such as ambient light, noise, and temperature are control variables, which are kept constant during the experiment for all participants. The assumption is that the variables under consideration have correlations and interrelationships and that these may be modeled if a sufficient volume of data is collected in an efficient manner.

**Fig. 4:**
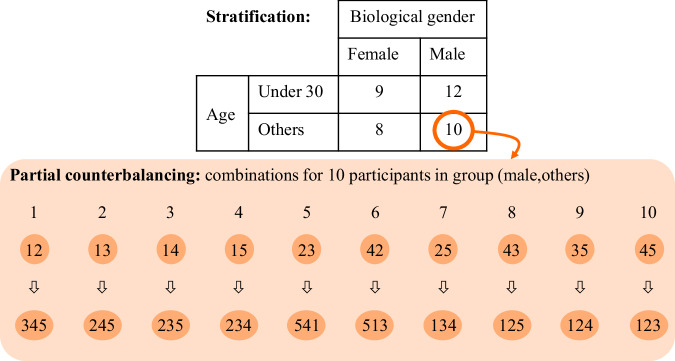
Handling covariates age and gender by stratified randomization.

From a design perspective, the study implements a repeated measures approach. Each participant is planned to drive five scenarios, efficiently capturing varied data from the same individual across different conditions. This dual-layered approach encompasses both within-subject and between-subject variations. The dataset’s target demographic comprises licensed drivers in Germany. Of the 50 initial participants, those prone to motion sickness are excluded from the test group, however their data are separately available. The test group consists of 39 participants. Balancing the sample size and covariates is deemed crucial, especially for small trials. The covariates age and gender are handled through the stratification method, assigning two levels to each (female or male and under or over 30 years old), resulting in four blocks based on these combinations, as shown in Fig. 4. To prevent carryover effects such as learning or practice effect, fatigue effect, and context effect, counterbalancing is applied across blocks. Given the substantial participant count needed for full counterbalancing, a partial counterbalancing approach is adopted. This ensures equal representation of each scenario in the first two or last three driven scenarios in every block. Each participant goes through the experiment only once, eliminating repetition. In summary, the experimental design is methodically constructed, addressing various factors and potential confounding variables, ensuring the validity and reliability of the gathered data.

### Driving scenarios

In the simulation, one familiarization drive and five unique driving scenarios are created. The primary aim of the familiarization drive is to give participants an opportunity to practice driving in the simulator, adjusting to the virtual environment before moving on to the main part. The objective of the main five driving scenarios is to induce different driver states, including the elicitation of distinct emotions. All of the main driving scenarios are composed of a sequence of events, subtly differentiated from each other to avoid learning effect. This variance among scenarios provides a rich spectrum of driver state factors for study. Tables 3–7 give an overview of the sequence of events for each scenario, including a duration range specified for each event. The duration of each event is different for each driver, depending on their manual driving speed, the time they change from manual to automated mode, and vice versa. The duration of the takeover event is a maximum of 10 s, which is the time budget of the TOR, and if the drivers do not react, an accident occurs and the scenario ends. The n-back games are set to have 30 s duration at each round, however this can also increase or decrease based on engagement of the drivers to the game. Some of the events are not present in the data collected from some participants because the conditions for the events are not met. For example, if the drivers do not switch to automated mode, they are not offered n-back games and data on these events are missing. In general, with the exception of the takeover situations, drivers were not forced to complete the specified tasks in order to create a more realistic ride, resulting in differences in the duration of task completion between individuals.

**Table 3:** Sequence of events in the scenario where no emotion is evoked.

Event label	SAE Level	NDRT	Event explanation	Duration range [s]
Attention objects	0	None	While driving, the following objects come one after the other: person/s standing on the right side of the road, a car coming from the opposite side on the opposite lane with the same speed as ego-vehicle, a bicycle coming from the opposite side on the opposite lane with 25% of the speed of the ego-vehicle, and a person crossing the road in front of the ego-vehicle, with *TTC* = 4 s.	0–60
	3	2-back game	N-Back game on tablet ready to be played if desired, with auditory and visual announcement.	0–60
Car-following	2	None	A vehicle from behind comes in front of the ego-vehicle and brakes three times with different situation criticalities, the *TTC* being 5.04 s, 10.08 s, and 15.12 s, respectively.	0–190
Car-following	2	Auditory digit-span level 1	A vehicle from behind comes in front of the ego-vehicle and brakes three times with different situation criticalities, the *TTC* being 5.04 s, 10.08 s, and 15.12 s, respectively.	0–190
	2	None	Automated system is at SAE L2, and drivers are expected to monitor what is happening on the road and intervene when necessary.	0–40
	3	None	Automated system is at SAE L3, and drivers can relax.	0–40
Dog on the road	3	None	A dog is loose on the opposite side of the road. The automated system detects this and reports it to the driver.	0–60
Sound/scene from outside	3	1-back game	N-Back game on tablet ready to be played if desired, with auditory and visual announcement. Meanwhile, sound of fans cheering on their team in stadium can be heard from outside in the distance.	0–50
Takeover situation	3 → 0	1-back game	Drivers play the game on their tablet while exposed to TOR due to a cat playing in front of ego-vehicle on the road and the system being unable to respond. The visual modality of TOR includes a clue to brake.	0–5
	0	Auditory digit-span with varying difficulty level	System prompts drivers to solve the auditory digit-span task while driving manually.	0–60

**Table 4:** Sequence of events in the scenario where anger is evoked.

Event label	SAE Level	NDRT	Event explanation	Duration range [s]
Attention objects	0	None	While driving, the following objects come one after the other: person/s standing on the right side of the road, a car coming from the opposite side on the opposite lane with the same speed as ego-vehicle, a bicycle coming from the opposite side on the opposite lane with 25% of the speed of the ego-vehicle, and a person crossing the road in front of the ego-vehicle, with *TTC* = 4 s.	0–110
Car-following	2	None	A vehicle from behind comes in front of the ego-vehicle and brakes three times with different situation criticalities, the *TTC* being 5.04 s, 10.08 s, and 15.12 s, respectively.	0–276
	3	2-back game	N-Back game on tablet ready to be played if desired, with auditory and visual announcement.	0–30
Sound/scene from outside	3	None	The police are looking for a blue car. The automated system reports this and asks the driver if the blue car can be seen in the vicinity.	0–257
	3	None	Automated system is at SAE L3, and drivers can relax.	0–60
Takeover situation	3 → 0	None	Drivers exposed to TOR due to a cow standing in front of ego-vehicle on the road and the system being unable to respond. The visual modality of TOR includes a clue to steer right.	0–10
	0	None	Manual driving in an empty road.	50–180
Sound/scene from outside	3	2-back game	N-Back game on tablet ready to be played if desired, with auditory and visual announcement. Meanwhile, a police car with active siren approaches the ego-vehicle and overtakes it from the left and drives on.	0–257
Car-following	2	Auditory digit-span level 2	A vehicle from behind comes in front of the ego-vehicle and brakes three times with different situation criticalities, the *TTC* being 5.04 s, 10.08 s, and 15.12 s, respectively.	0–276
Takeover situation	3 → 0	Subway surfers game	Drivers play game on the tablet while exposed to TOR due to a construction on the road and the system being unable to respond.	0–10

**Table 5:** Sequence of events in the scenario where surprise is evoked.

Event label	SAE Level	NDRT	Event explanation	Duration range [s]
Attention objects	0	None	While driving, the following objects come one after the other: person/s standing on the right side of the road, a car coming from the opposite side on the opposite lane with the same speed as ego-vehicle, a bicycle coming from the opposite side on the opposite lane with 25% of the speed of the ego-vehicle, and a person crossing the road in front of the ego-vehicle, with *TTC* = 4 s.	0–120
	3	2-back game	N-Back game on tablet ready to be played if desired, with auditory and visual announcement.	0–60
Sound/scene from outside	3	None	A tollgate is ahead without any officers. The automated system reports this to the driver and announces that he will drive through the gate.	0–298
Car-following	2	None	A vehicle from behind comes in front of the ego-vehicle and brakes three times with different situation criticalities, the *TTC* being 5.04 s, 10.08 s, and 15.12 s, respectively.	0–93
	3	None	Automated system is at SAE L3, and drivers can relax.	0–60
Takeover situation	3 → 0	None	Drivers exposed to TOR due to lane marking recognition errors resulting in possible crashes with roadway structures.	0–10
Car-following	2	Auditory digit-span level 3	A vehicle from behind comes in front of the ego-vehicle and brakes three times with different situation criticalities, the *TTC* being 5.04 s, 10.08 s, and 15.12 s, respectively.	0–93
Sound/scene from outside	3	3-back game	N-Back game on tablet ready to be played if desired, with auditory and visual announcement. Meanwhile, a police siren can be heard as an accident has occurred on the opposite side of the road and police cars are on the scene.	0–585
Takeover situation	3 → 0	3-back game	Drivers play the game on their tablet while exposed to TOR due to a live accident happening directly in front of the ego-vehicle. The visual modality of TOR includes a clue to steer left.	0–10
	0	None	Manual driving with low traffic density.	0–60

**Table 6:** Sequence of events in the scenario where sadness is evoked.

Event label	SAE Level	NDRT	Event explanation	Duration range [s]
Attention objects	0	None	While driving, the following objects come one after the other: person/s standing on the right side of the road, a car coming from the opposite side on the opposite lane with the same speed as ego-vehicle, a bicycle coming from the opposite side on the opposite lane with 25% of the speed of the ego-vehicle, and a person crossing the road in front of the ego-vehicle, with *TTC* = 4 s.	0–90
Sound/scene from outside	3	None	Some containers are placed on the right side of the road, which are not recognized by the sensors of the automated system. The system reports this to the driver and continues the journey.	0–298
	3	None	Automated system is at SAE L3, and drivers can relax.	0–30
	3	2-back game	N-Back game on tablet ready to be played if desired, with auditory and visual announcement.	0–30
Car-following	2	None	A slow vehicle in front of the ego-vehicle brakes three times with different situation criticalities, the *TTC* being 5.04 s, 10.08 s, and 15.12 s, respectively.	0–202
Takeover situation	3 → 0	Reading book	Drivers are reading a book they are holding while exposed to the TOR due to a group of horses crossing the road and the system being unable to respond.	0–7
	0	None	Manual driving at an intersection where the ego vehicle has the right of way. The vehicle in the opposite lane turns (its) left and the vehicle on the right turns (its) right, both before the ego vehicle arrives.	0–60
	0	None	Manual driving in an empty road.	0–60
Car-following	2	Auditory digit-span level 4	A slow vehicle in front of the ego-vehicle brakes three times with different situation criticalities, the *TTC* being 5.04 s, 10.08 s, and 15.12 s, respectively.	0–202
Sound/scene from outside	3	4-back game	N-Back game on tablet ready to be played if desired, with auditory and visual announcement. Meanwhile, music can be heard from outside as a group camps on the roadside.	0–298

**Table 7:** Sequence of events in the scenario where fear is evoked.

Event label	SAE Level	NDRT	Event explanation	Duration range [s]
Attention objects	0	None	While driving, the following objects come one after the other: person/s standing on the right side of the road, a car coming from the opposite side on the opposite lane with the same speed as ego-vehicle, a bicycle coming from the opposite side on the opposite lane with 25% of the speed of the ego-vehicle, and a person crossing the road in front of the ego-vehicle, with *TTC* = 4 s.	0–70
	3	None	Automated system is at SAE L3, and drivers can relax.	0–60
Car-following	2	None	A vehicle in front of the ego-vehicle brakes three times with different situation criticalities, the *TTC* being 5.04 s, 10.08 s, and 15.12 s, respectively.	0–99
Sound/scene from outside	3	None	A tree has fallen on the road and is blocking the way for road users. The system reports this to the driver and changes the route.	0–255
	3	2-back game	N-Back game on tablet ready to be played if desired, with auditory and visual announcement.	0–30
Car-following	2	Auditory digit-span level 5	A slow vehicle in front of the ego-vehicle brakes three times with different situation criticalities, the *TTC* being 5.04 s, 10.08 s, and 15.12 s, respectively.	0–99
Sound/scene from outside	3	5-back game	N-Back game on tablet ready to be played if desired, with auditory and visual announcement. Meanwhile, the flying wild night birds can be heard from outside.	0–255
Takeover situation	3 → 0	Pressing communication pedal with right foot	Drivers are asked to press a communication pedal near their right foot when they see deer, as they are exposed to the TOR due to a group of horses crossing the road and the system being unable to respond.	0–7
	0	Answering phone on loud speaker	Manual driving in an empty road while speaking on the phone.	0–40

Each of the five specialized scenarios are designed to evoke a specific emotion, namely: no emotion, anger, surprise, sadness, and fear. Except the scenario with no emotion induction, the other four scenarios are divided into two segments. In the first segment, a particular emotion - anger, surprise, sadness, or fear - is evoked and maintained. The first segment of all scenarios starts in manual driving mode, where a pedestrian on the roadside, a car and a bicycle coming from the opposite side, and a person crossing the road are included as attention objects in the design. The other planned events in the emotional segment of the scenarios are car-following in SAE L2 with a slow car driving ahead, automated driving in SAE L3 with no task for the driver, SAE L3 combined with playing a 2-back game on the NDRT tablet, and playing auditory stimuli from outside the vehicle to attract drivers’ attention. The sequence of these events is selected based on the characteristics of the road in the simulation map, and with the exception of the attention objects at the beginning, the order of the other events is not the same in the different scenarios.

The second segment of each scenario is designed to create an emotionally neutral setting where no explicit emotional provocation occurs. This segment includes additional events such as TOR, playing auditory digit-span task, and speaking on the phone. Each scenario has been carefully designed with its own unique characteristics yet maintaining enough similarities with the other scenarios to ensure comparability of the gathered data. The “no emotion” scenario serves as a control, with no specific emotional state being induced, thereby establishing a baseline. On the other hand, the remaining four scenarios seek to elicit varying emotional states through different means. For instance, the use of emotional video clips prior to the start of the drive, as well as the implementation of monotonous and even-driven methods during the drives, have been employed to trigger the desired emotional responses^31^. Details on the design of each scenario are described below. The scenarios are named after the emotions that are to be evoked by emotional elements in the scenarios.

#### Familiarization

The familiarization drive is set along a loop of serene country roads, devoid of traffic lights or other traffic members, thereby ensuring an accident-free practice environment. The virtual drive takes place in the midday hours to provide clear visibility and allow participants to focus solely on mastering the operation of the simulator.

#### No emotion

In this scenario, no specific emotion is elicited from the participants. This drive also takes place during the midday, providing a neutral environment. During this scenario, participants are initially provided with 2-back game, which then reduces to 1-back game, giving them an activity that requires low cognitive load. When a TOR is presented in this scenario, it is supplemented with a visual reaction cue for drivers: a text reading “Brake” appears in red on the lower left side of the main display, near the navigation instructions, serving as an additional prompt for the driver (see Fig. 3c). Lastly, an auditory digit-span task is played towards the conclusion of the scenario, with its difficulty level fluctuating between one and five.

#### Anger

The scenario is designed to be driven with anger emotion. To induce this emotion, before the driving session commences, drivers watch a short clip (6′ 7″) from the film “Seven” selected from FilmStim database^32^ which is assumed to evoke anger feeling. The scenario is set in midday. Throughout the drive, participants are given n-back game and auditory digit-span task at difficulty level 2. TOR is exposed to drivers two times within this scenario. First time TOR has also a reaction clue in the form of a red arrow to right (see Fig. 3c). Second time, in the last part of the drive, the drivers are asked to play the Subway Surfers game when a simple TOR is exposed to them.

#### Surprise

The driving scenario is specifically designed to elicit a state of surprise in its first part. To accomplish this, several unexpected events are planned at the beginning of the drive. These include a sudden snowfall during sunny weather, a pedestrian who abruptly crosses the road and immediately retraces their steps, followed by an abrupt end of the snowfall^31^. This sequence of unexpected and rapidly changing events helps create a sense of surprise. As for cognitive tasks, the n-back game begins at difficulty level 2 and then escalates to level 3. The auditory digit-span task played during the drive has difficulty level 3. In the last part a TOR is presented, supplemented with a visual clue in the form of a red arrow pointing to the left, positioned on the lower left side of the main display near the navigation instructions (see Fig. 3c).

#### Sadness

This scenario is crafted to evoke and sustain a feeling of sadness in the participants in its first part. To instigate this emotion, a short clip (4′ 25″) from the film “City of angels” selected from FilmStim database^32^ is played for the drivers before the drive commences, which has been identified as effective in eliciting sadness. In addition, the music piece “Adagio for Organ and Strings in G Minor” plays during the first part of the drive until end of first car-following event, to help maintain the induced emotional state^33,34^. Furthermore, the rainy weather and dark clouds serve to augment the melancholy atmosphere. As for cognitive challenges, the n-back game begins at difficulty level 2 and then escalates to a more demanding level 4, and the auditory digit-span task is also presented at difficulty level 4. In some part of the scenario drivers are asked to read a book aloud. As the drivers’ hands are engaged with holding the book, a simple TOR is introduced due to a group of horses crossing the road.

#### Fear

To trigger fear, a short clip (4′ 25″) from the film “The shining” selected from FilmStim database^32^ is played to the drivers before the drive begins. This clip has been identified as effective in eliciting the sense of fear. The setting is further enhanced by playing “A night on the bare mountain” music during the initial part of the drive until beginning of second car-following to sustain the induced fear^33^. To intensify the fearful ambiance, the drive is set under dark daytime conditions, compelling drivers to switch on the vehicle’s headlights to navigate their path. As for cognitive tasks, the n-back game initiates at difficulty level 2 and then progresses to an advanced level 5, and the auditory digit-span task is held at difficulty level 5, providing high cognitive load. A unique task in this scenario requires drivers to press a communication pedal every time they spot a deer. Simultaneously, a simple TOR is issued when a deer stands in the path of the vehicle.

### Computational processing

All data except videos are provided at a sampling rate of 256. However, if the data from a particular sensor is to be used separately, it can be downsampled to the acquisition frequency of the sensor. The videos provided have a sampling rate of 30 FPS.

Synchronization is carried out directly during data recording using the SCANeR studio real-time simulation software. This software has specific interfaces for the BIOPAC sensor, camera, and SmartEye system that enable reliable connection and data synchronization. In addition, the simulation software offers a communication package for self-defined communication with other sensors, which is used in this work to realize the synchronized data stream of Empatica E4 wristband and BodiTrak seat-pressure-sensor mats.

#### Environment data

Data from the driving environment is collected by SCANeR studio real-time software that initially captures this information at a rate of 20 and resamples to 256 to enhance synchronization. A vital part of the data assessment focuses on the immediate vicinity of the ego-vehicle. When an object is detected in the same lane ahead of the ego-vehicle, distance to object (*DTO*) is calculated as

1 $$DTO=\sqrt{{\left({x}_{{\rm{ego}}}-{x}_{{\rm{front}}}\right)}^{2}+{\left({y}_{{\rm{ego}}}-{y}_{{\rm{front}}}\right)}^{2}}\,[{\rm{m}}],$$

where (*x*_ego_, *y*_ego_) and (*x*_front_, *y*_front_) denote position of the ego-vehicle and the object ahead, respectively. Furthermore, the time to collision (*TTC*) with any approaching object or pedestrian is determined as

2 $$TTC=\frac{DTO}{{\nu }_{{\rm{ego}}}-{\nu }_{{\rm{front}}}}\;[{\rm{s}}],$$

where *v*_ego_ and *v*_front_ are speed of ego-vehicle and front vehicle, respectively.

#### Video

The video data is recorded using an Intel RealSense D435 camera connected to the video module of the SCANeR software, to ensure synchronization with the remaining data. The videos are captured at a frame rate of 30 FPS. While the original frames are recorded at a size of 640 × 480 px and encompass a larger area, the provided videos in the dataset have been cropped to focus specifically on the face. These cropped frames have a smaller dimension of 200 × 200 px using the OpenCV library^35^. The resulting videos, centered on facial expressions and reactions, are then saved in the widely compatible MP4 format, facilitating convenient viewing and analysis. The illumination of the driving scenarios is not the same, i.e. three of the scenarios take place during the day, one at night, and one in the rain, resulting in different lighting conditions in the captured videos. However, the data is not preprocessed further to keep it as much raw as possible.

#### Eye tracking

Eye tracking data is collected at frequency of 120. It encompasses the Percentage of Eyelid Closure (*PERCLOS*), which is determined from

3 $$PERCLOS=\frac{EO}{\max \{E{O}_{1},E{O}_{2},\ldots ,E{O}_{n}\}}\ast 100\;[ \% ],$$

where *EO* denotes eyelid opening and *n* in the length of the data. To ensure that the eye tracking information aligns seamlessly with remaining data, it is subsequently resampled to 256. During the resampling transition, the previous sensor readings are held constant until the new reading arrives to maintain continuity of data.

#### PPG/EDA

The Empatica E4 wristband sensor, worn on the participant’s dominant arm, collects data regarding wrist acceleration and various physiological indicators. The gathered data is preprocessed, resampled at a rate of 256, and provided in text files.

Acceleration data from the Empatica device is measured within a range of ±2 *g*, and the sensor’s output is quantified in units of 1/64 g. However, the acceleration data in manD 1.0^26^ is already converted, rendering the unit of acceleration data as m/s^2^. The original sampling rate of the acceleration data is 32.

*BVP* is captured with units in nanoWatt (nW) at a sampling rate of 64. The Empatica measures *BVP* within a range of 500. From this *BVP* data, the instantaneous *HR* is calculated and presented under the column “Empatica/HR”. This is achieved by identifying the peaks in the *BVP* using the scipy.signal package^36^, taking 240 BPM as the maximum acceptable *HR*. The *HR* is then calculated following

4 $$HR=60\ast \frac{{\rm{Samplingrate}}}{BV{P}_{{\rm{peak}}}^{i}-BV{P}_{{\rm{peak}}}^{i-1}}\;[{\rm{BPM}}],$$

where the sampling rate is 256 and $BV{P}_{{\rm{peak}}}^{i}$ shows the *i*^th^ peak of the *BVP*. *EDA* is sampled at 4, with the unit of measurement being microSiemens (S). The *EDA* data is then used to derive skin conductance level (*SCL*) and skin conductance response (*SCR*) values using the neurokit2 library^37^. These calculated values are included in the dataset under the columns “Empatica/SCL” and “Empatica/SCR”.

The sensor also captures peripheral skin temperature data of the participants, sampled at 4 and provided in degrees Celsius (°C).

#### EEG/ECG

The BIOPAC sensor system captures data through nine *EEG* channels and a single *ECG* channel, offering dynamic range of 1000. The system operates at a sampling rate of 256. According to the manufacturer’s specifications, the BIOPAC system provides a reported accuracy and resolution of 3.0 peak-to-peak and 0.038 resolution for *EEG* signals, and 3.0 peak-to-peak with a resolution of 0.06 for *ECG* signals. Initial signal processing is performed using the AcqKnowledge 4 software, which applies a 0.1 highpass filter and a 67 lowpass filter. Upon acquisition, a data preprocessing step is implemented to correct bad channels; channels presenting extreme average values are detected and interpolated using the average of neighboring channels. If all neighboring channels are similarly compromised, the defective channel is removed. Channels which exhibit a frozen (constant) value are also detected, and these unchanging segments are interpolated using the average of neighboring channels. In cases where all neighboring channels are similarly frozen, the unchanging segment of the channel is removed. In all readings, the initial 6000 steps (about 24 seconds) are discarded as the sensor requires this time to begin accurate data capture. No further preprocessing is applied to the data to keep them as much row as possible and allow the users of the dataset to apply their own data analysis and artifact removal techniques.

Instantaneous *HR* is also estimated based on *ECG* data by first calculating the *IBI* by identifying peaks in the *ECG* signal using the scipy.signal package, with a defined maximum *HR* of 240. *HR* is then computed according to Eq. 4. The “BIOPAC/HR” column demonstrates the calculated *HR* at a sampling rate of 256; each time a heartbeat is detected, the *HR* value is updated accordingly. The HR obtained from the ECG data of the BIOPAC sensor can be used as a reference compared to the instantaneous HR obtained by Empatica, because the ECG is acquired with electrodes placed directly on the heart area, so the data is acquired with less delay and noise.

#### Seat pressure distribution

The BodiTrak2 Pro seat-pressure-sensor mats employ pressure sensors for data acquisition at a sampling rate of 15 FPS. Despite the initial acquisition rate, the provided data is structured in an excel file at a higher sampling rate of 256 FPS synchronized with other provided data. Following data acquisition, a preprocessing phase is implemented to correct any misrecordings. Frames that are entirely zero, potentially due to network failure, are corrected by refilling them with the values from the preceding frame. Beyond the excel data file, heatmaps illustrating pressure distributions on the seat and backrest of drivers are separately provided as images and videos in PNG and MP4 formats, respectively. In creating these videos, the data are initially downsampled to 30, and a Gaussian filter is applied to smooth the data utilizing the scipy.ndimage package. Subsequently, images are plotted based on the smoothed data, and the OpenCV library is used to create videos from these images. As a result, videos of heatmaps that depict the pressure on the seat and backrest of drivers are supplied separately in MP4 format, providing a visual representation of the pressure dynamics over time.

#### NDRT

During the experiment, data related to NDRTs are gathered to provide comprehensive insights. Specifically, n-back data are collected separately using PsyToolkit^38^, a specialized toolkit tailored for the demonstration, programming, and execution of cognitive-psychological experiments. Ensuring congruence across datasets, the n-back data is then synchronized with other information at a consistent sampling rate of 256. Similarly, data derived from the digit-span task and the Subway Surfers game are also synchronized, matching the same sampling rate.

## Data Records

**Fig. 5:**
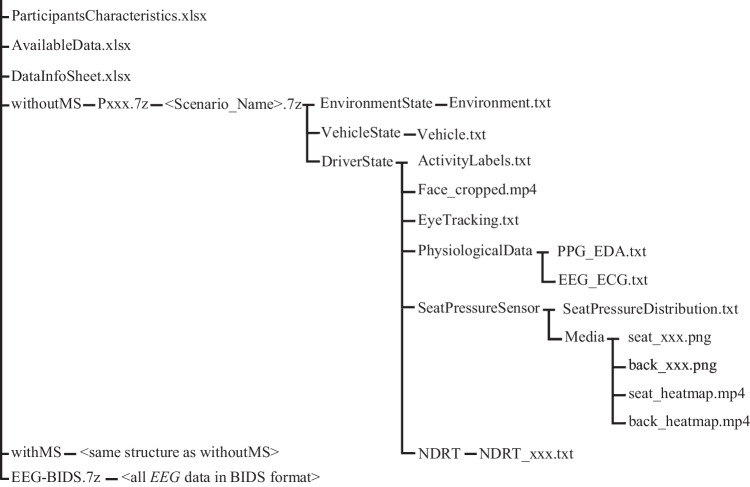
Structure of the provided dataset.

The open access data is available for all researchers via the Harvard Dataverse repository manD 1.0^26^. The entire dataset can be downloaded either in full or partially, according to the researcher’s requirements. The data is organized into different levels, as depicted in Fig. 5. At the root of the repository, four spreadsheets, a BIDS structure, and a python file are available:

- “AvailableData.xlsx”, contains a report on the data availability in relation to the participant, the driving scenario, and the sensor. In addition, this file contains information about the sequence of scenarios driven by the participants.
- “DataInfoSheet.xlsx”, gives information about the data points contained in each file, including explanation, unit, range, and interpretation of the values.
- “DESResults.xlsx”, outlines the Z-Score of the DES assessments completed by participants before and after driving each scenario.
- “ParticipantsCharacteristics.xlsx”, contains information about the characteristics of the participants, including biological gender, age, height, BMI, sight correction, years of driving experience, annual driving distance, and experience with assistance systems.
- “EEG-BIDS.7z” provides the *EEG* data gathered from all participants in a single brain imaging data structure (BIDS)^39,40^. BIDS is a standard format for brain imaging data, the use of which is promoted by the neuroimaging community.
- “DataExtraction.py” consists of functions for the extraction of files with 7z format, the derivation of *HR* from *ECG* and *BVP*, and the creation of heatmaps from pressure sensor readings.

Data gathered from participants who experienced motion sickness during the experiment (withMS) and those who did not (withoutMS) are provided in two separate directories, each having the same structure. However, the number of participants who experienced motion sickness is lower and the data gathered from them is very limited, e.g., only data gathered during the familiarization drive until they clearly felt symptoms of motion sickness such as nausea and dizziness.

Each of the directories includes zipped folders of related participants (P1.7z, P2.7z, …). Each subject comprises one or more familiarization drives and five driving scenarios, which are also compressed in the dataset. Lastly, the data from each scenario is organized into three modules: “EnvironmentState”, which contains data about the surrounding situation and other traffic members provided by the simulation software; “VehicleState”, which contains data about the driving dynamics of the ego-vehicle, again given by the simulation software; and “DriverState”, which contains data about the driver gathered from all sensors.

Data in the DriverState module is further detailed into data gathered from each sensor, including physiological data, seat pressure distributions, eye tracking information, camera videos, NDRT performances, and activity labels. Below is a brief explanation of the data files included in the dataset.

### xxxMS/Pxxx/<Scenario_Name>/ EnvironmentState/EnvironmentState.txt

This file provides a comprehensive snapshot of various parameters associated with the driving environment. This file captures information on prevailing weather conditions, specifics of the road including speed limits, and the relative distance to surrounding vehicles. The file also details the lateral shift, speed, and acceleration metrics of nearby vehicles. Additionally, it registers lane-crossing actions of front vehicles and provides crucial data about any objects or pedestrians ahead, including their distance and speed. Another metric included is the *TTC* which gives information about situation criticality. Beyond these data points “EnvironmentalEvent” column categorizes and labels the environmental scenarios, ranging from “no event” situations to specific events like attention objects, car-following, sound/scene from outside, dog on the road, takeover situation, and accident of ego-vehicle. This file ensures that every aspect of the driving environment is meticulously documented for comprehensive analysis.

### xxxMS/Pxxx /<Scenario_Name > / VehicleState/VehicleState.txt

The VehicleState.txt file contains the recorded essential dynamics data, like speed, acceleration, lateral shifts, and lane crossing information. The file also provides insights into the SAE automation level of the vehicle, logs background music being played, and any interaction signals presented to the driver, which might influence their driving behavior or decision-making.

### xxxMS/Pxxx /< Scenario_Name > / DriverState/Face_cropped.mp4

Face_cropped.mp4 serves as a visual record of drivers’ facial expressions during their driving experience. Captured from a camera directed at driver’s face, this file offers insights into the real-time emotional and cognitive facial responses of drivers as they navigate various road situations. Each video clip has a resolution of 200 × 200 px and is recorded at a rate of 30 FPS.

### xxxMS/Pxxx /< Scenario_Name>/ DriverState/ActivityLabels.txt

The ActivityLabels.txt file stands as a detailed record of both driving and non-driving activities undertaken by drivers throughout the experiment. Generated using simulator markings, this file depicts the spectrum of actions and behaviors exhibited by drivers, providing a foundation for understanding their engagement and responses during the study. It is planned to refine and enhance the granularity of these labels in future works.

### xxxMS/Pxxx /<Scenario_Name>/ DriverState/EyeTracking.txt

The EyeTracking.txt file delves into the dynamics of drivers’ ocular movements and focus during driving. A range of parameters is recorded in this file, beginning with the driver’s head position and rotation, offering insights into their overall orientation, specific eye movements like fixations (periods when the eyes are relatively stationary and gather information) and saccades (rapid eye movements between fixation points)^41^. Furthermore, it registers instances of blinks, providing data on the PERCLOS. The file also contains the pupil diameter, a potential indicator of cognitive load or emotional state. Additionally, the file identifies and logs the specific object or point the driver is looking at, offering a direct glimpse into their focus and attention. This combination of data helps to understanding the driver’s alertness, engagement, and possible distractions.

### xxxMS/Pxxx /<Scenario_Name>/ DriverState/PhysiologicalData/PPG_EDA.txt

This file consists of physiological data captured using the Empatica E4 wristband, which drivers put on their dominant hand’s wrist. The file captures the acceleration of the wrist, giving insights into the driver’s hand movements, which can be used to improve data filtering or estimate possible gestures. Moreover, *BVP* and *EDA*, two pivotal markers of physiological arousal are recorded in this file. From these raw data, other crucial metrics are derived: *HR* is extracted from the *BVP*, while *SCL* and *SCR* are deduced from the *EDA*. Additionally, the file logs the skin temperature, potentially shedding light on stress levels and comfort.

### xxxMS/Pxxx /<Scenario_Name>/ DriverState/PhysiologicalData/EEG_ECG.txt

The EEG_ECG.txt file provides an insight into the neural and cardiac activities of individuals, captured using the BIOPAC sensor system. This file primarily encompasses data from nine *EEG* channels - namely *Poz, Fz, Cz, C3, C4, F3, F4, P3*, and *P4* - each of which maps to specific regions of the brain. These channels record the electrical activity of the brain, shedding light on cognitive processes and states of arousal. Complementing the *EEG* data is the *ECG* information, which charts the electrical activity of the heart. Also from the *ECG* data the *HR* is extracted, offering a direct measure of cardiac activity. The EEG_ECG.txt file, with its combination of neural and cardiac data, stands as a precise tool and reference for understanding both cognitive and physiological responses in real-time scenarios.

### xxxMS/Pxxx /<Scenario_Name>/ DriverState/SeatPressureSensor/SeatPressureDistribution.txt

**Fig. 6:**
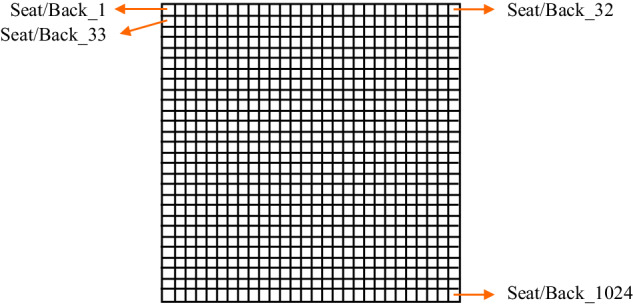
Numbering of seat-pressure sensors.

SeatPressureDistribution.txt file consists of time-stamped pressure-sensor readings from both the seat and backrest of drivers. Each frame contains 2048 readings per frame (structured as 2 × 32 × 32), to represent data from distinct pressure sensors. The columns within the file are distinctly labeled as Seat_xxx and Back_xxx for seat and backrest readings, respectively. The numbering and positioning of these sensors are visually illustrated in the Fig. 6.

### xxxMS/Pxxx /<Scenario_Name>/ DriverState/SeatPressureSensor/Media/seat_xxx.png and back_xxx.png

The files seat_xxx.png and back_xxx.png offer a visual representation of pressure distribution on the two dedicated seat-pressure-sensor mats. These images, sampled at a frequency of 30 FPS, provide a real-time snapshot of how drivers adjust and shift their weight, allowing for an intuitive understanding of their comfort, positioning, and movements.

### xxxMS/Pxxx /<Scenario_Name>/ DriverState/SeatPressureSensor/Media/seat_heatmap.mp4 and back_heatmap.mp4

Last components of pressure distribution are the video files seat_heatmap.mp4 and back_heatmap.mp4 with 30 FPS, visualizing the pressure distribution on the seat and backrest over time. These videos are compiled directly from the pressure distribution images housed in the same folder, offering a sequential visualization of driver’s posture and movements throughout the driving experience.

### xxxMS/Pxxx /<Scenario_Name>/ DriverState/NDRT/NDRT_xxx.txt

Three NDRT_xxx.txt files provide detailed information, all timestamped, about NDRTs centered around various games. The NDRT_nback.txt captures data on the n-back game. It details the block number ranging from one to four, with each block containing between 25 to 30 digit exposures. Alongside, it records the user’s performance metrics such as score, matches, misses, false alarms, and reaction time. Additionally, the game difficulty level is also documented. The NDRT_Digit_Span.txt file is a report of the digit-span task. It captures timestamps, the playing status marked as ‘Digit_Span_Playing’ column (which turns 1 when the user is actively engaged in the game), the game difficulty level, and user responses which are marked 1 for correct answers and −1 for incorrect ones. Lastly, the NDRT_SubwaySurfers.txt is more streamlined, noting timestamps and the user’s playing status for the game Subway Surfers. Here, 1 indicates active gameplay, while 0 signifies inactivity. This file doesn’t delve into the performance metrics of the game, focusing solely on play engagement.

## Technical Validation

In the present contribution, the technical validation included two steps: quality control to check the availability and reliability of the data and experimental validation to check the scope of the data.

### Quality control of collected dataset

**Fig. 7:**
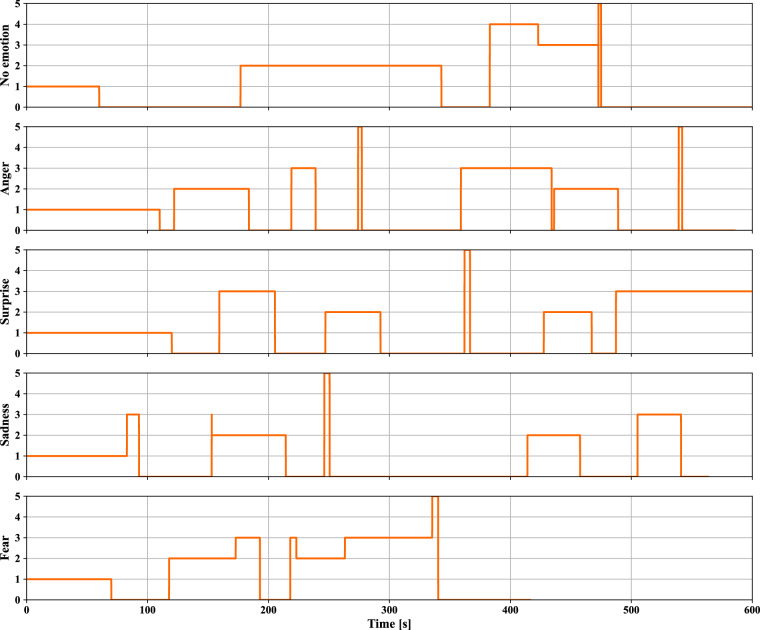
Sample event sequences for the driving scenarios. 0: no event; 1: attention objects; 2: car-following; 3: sound/scene from outside; 4: dog on the road; 5: takeover situation.

In the dataset, certain data points are absent due to technical problems. To provide clarity on this, the “DataAvailability.xlsx” file offers an overview of the available data. For environmental data, both the range of data and the sequence of the recorded environmental events are rigidly monitored. Fig. 7 shows example sequences of events for the 5 scenarios.

**Fig. 8:**
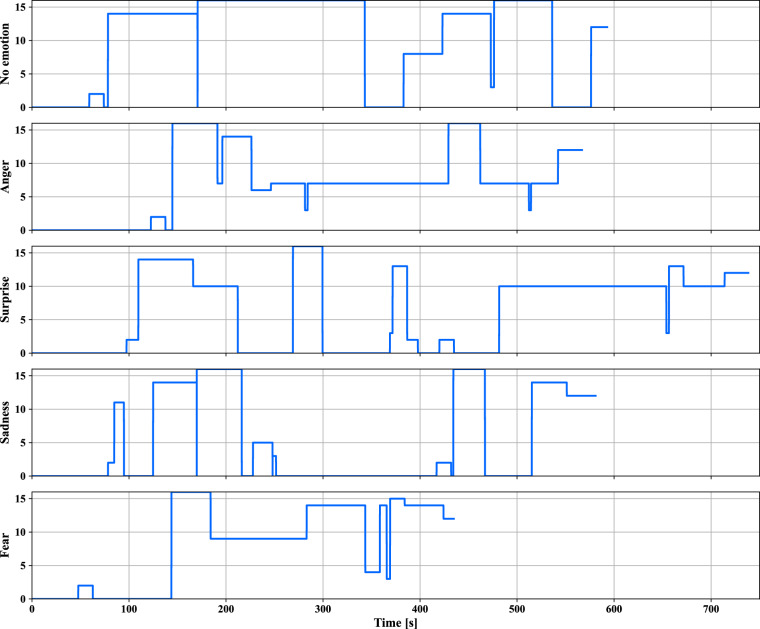
Example sequence of interaction signals. 0: no signal; 1: I don’t know; 2: autonomous mode available; 3: TOR; 4: engagement of communication pedal; 5: read the book; 6: looking for a car; 7: car found; 8: a dog on the road; 9: unknown object on the road; 10: a tollgate ahead; 11: unknown objects on the road side; 12: end of the ride; 13: an accident detected; 14: game available on the iPad; 15: phone call; 16: auditory digit-span task.

The vehicle data has also undergone the validation. Specifically, the order of interaction signals (see Fig. 8), automation mode, and plots of specific vehicle dynamics parameters, such as travelled distance, have been verified.

One of the unique attributes of the driver activity data is its multi-labeling feature, signifying that multiple activities can occur simultaneously. Within the validation process the plausibility of these overlaps are examined.

A few participants opted not to include their videos in the dataset. To further ensure the dataset’s integrity, both face videos and pressure heatmap videos are played back to confirm that they are correctly encoded, contain the requisite information, and are free from playback issues.

In the realm of eye tracking data, any unavailable column is excised. Supplementary columns are provided for the confidence of the head position, head rotation, and pupil diameter. These columns represent the average data quality, with values spanning from zero to one, given directly from the sensor.

**Fig. 9:**
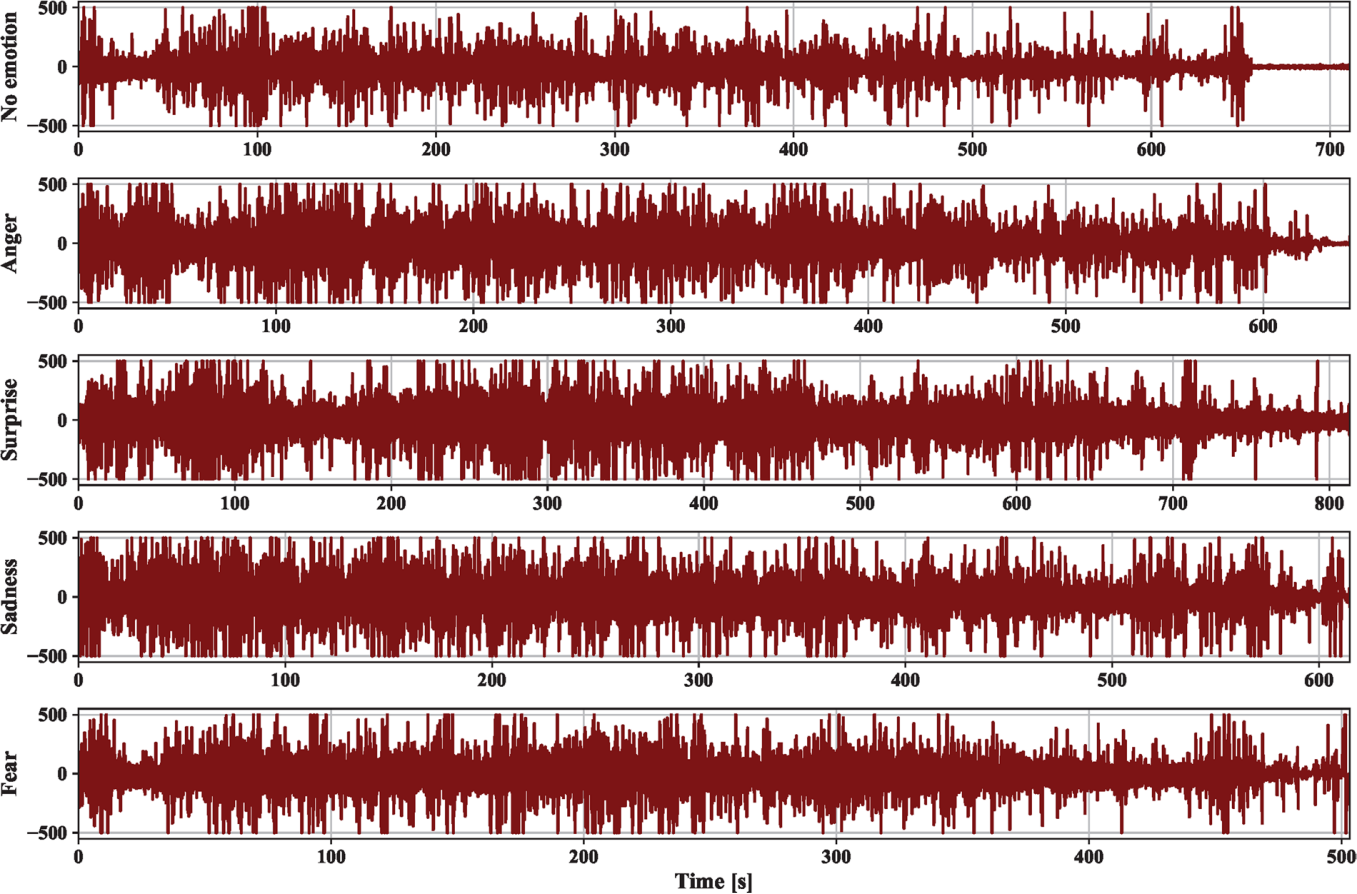
*BVP* [μV] data collected from all participants using Empatica E4 during five driving scenarios.

**Fig. 10:**
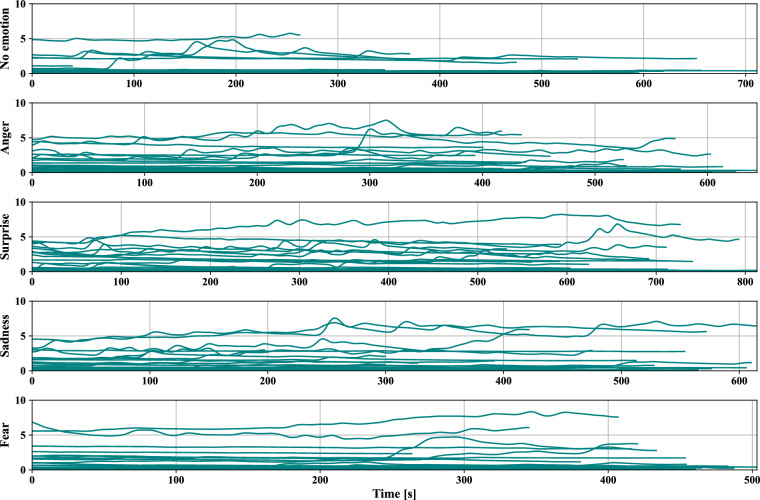
*SCL* [μS] data of all participants during five driving scenarios extracted from the EDA.

**Fig. 11:**
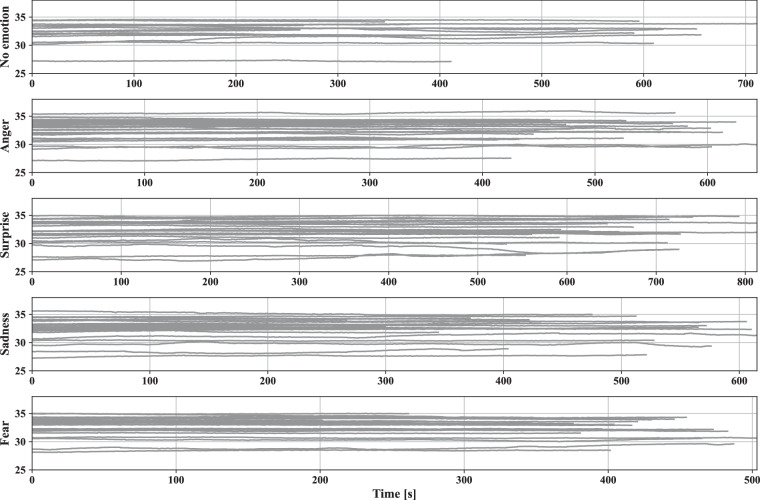
Skin temperature [°*C*] of all participants measured with Empatica E4 during five driving scenarios.

The physiological data, procured using the Empatica E4, has been validated by checking the data range for parameters such as *BVP*, *SCL*, and Skin temperature. Figs. 9–11 depict the range of gathered data.

When collecting *EEG* data, before start of the study the resistance between each electrode and scalp of each participant is checked to be less than 80, which shows good quality of connection and data collection according to the manufacturer. After the experiment, for the *EEG* and *ECG* datasets, the availability of channels has been confirmed and the pattern of the *ECG* data is controlled. The primary check was visual observation to ensure that the heartbeats are recognizable and clear anomalies such as constant values or white noise are removed. Later, during the extraction of the *HR* from the *ECG* data, the data is checked again by means of a code to make the same corrections if required.

The seat-pressure-sensor mats data is investigated for pressure reading ranges, and any void frames have been populated with preceding frame values.

Engagement with infotainment is confined to either the n-back task or the Subway Surfers game. Thorough checks are implemented to ensure the “Infotainment_Engagement” activity label accurately covers the timestamps when these games are in play, and that there’s no overlapping between the two.

By leveraging video data and following detailed noted protocols, there’s an ambition to craft more nuanced and refined activity labels in the future. This evolution aims to allow future analyses to delve deeper, offering richer insights into behavioral patterns.

### Experimental validation

**Fig. 12:**
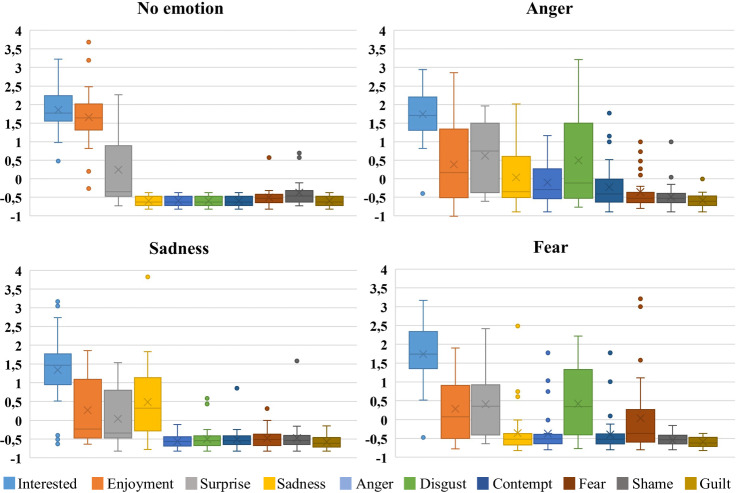
Z-Score [] of participants’ subjectively rated emotions (DES) immediately before the start of the driving scenarios.

A variety of effects and events were incorporated into the driving scenarios to vary the driver state. One element to elicit different emotions in participants was to play emotional video clips before the start of the three scenarios to elicit the emotions of anger, sadness, and fear. Participants were instructed to watch the video clips, then complete the DES, and drive off. After a one-way ANOVA test^42^, significant differences were found regarding the average of the rated emotions, enjoyment (*F*(4, 145) = 11.008, *P* = 7.9 E-08), surprise (*F*(4, 145) = 4.88, *P* = 1.0E-03), sadness (*F*(4, 145) = 13.86, *P* = 1.3E-09), anger (*F*(4, 145) = 9.17, *P* = 1.2E-06), disgust (*F*(4, 145) = 18.93, *P* = 1.5E-12), contempt (*F*(4, 145) = 4.44, *P* = 2.1E-03), and fear (*F*(4, 145) = 5.16, *P* = 6.4E-04), across all driving scenarios, where *F* denotes the ratio of between group variation and within group variation and *P* is the probability of obtaining an F-ratio as large or larger than the one observed, assuming that the null hypothesis of no difference amongst group means is true. Fig. 12 shows subjective ratings of participants. Post hoc analyses (T-test^43^) reveal that all three video clips evoked intended emotions as well as other emotions. Table 8 shows the significant (*P* < 0.05) emotions elicited by each video clip. This analysis does not control for the increase in the familywise error rate in the reported statistical analyses. Therefore, replication is recommended.

**Table 8:** Post-hoc analysis of significant emotions evoked by video clips. *m*: mean, *SD*: standard deviation.

Video	Evoked emotions					
	Enjoyment	Sadness	Anger	Disgust	Contempt	Fear
(No emotion as reference)	*m*: 1.66	*m*: −0.60	*m*: −0.60	*m*: −0.60	*m*: −0.60	*m*: −0.51
	*SD*: 0.96	*SD*: 0.21	*SD*: 0.35	*SD*: 0.93	*SD*: 0.20	*SD*: 0.29
Seven	*m*: 0.39	*m*: 0.04	*m*: −0.10	*m*: 0.50	*m*: −0.23	
	*SD*: 0.99	*SD*: 0.74	*SD*: 0.62	*SD*: 1.11	*SD*: 0.57	
	*P*: 7.1E-06	*P*: 1.8E-04	*P*: 2.5E-04	*P*: 3.8E-05	*P*: 5.8E-03	
City of angels	*m*: 0.28	*m*: 0.49				
	*SD*: 0.84	*SD*: 0.96				
	*P*: 3.0E-07	*P*: 2.0E-06				
The shining	*m*: 0.29			*m*: 0.42		*m*: 0.04
	*SD*: 0.88			*SD*: 0.89		*SD*: 0.98
	*P*: 4.7E-07			*P*: 1.3E-06		*P*: 1.4E-02

**Fig. 13:**
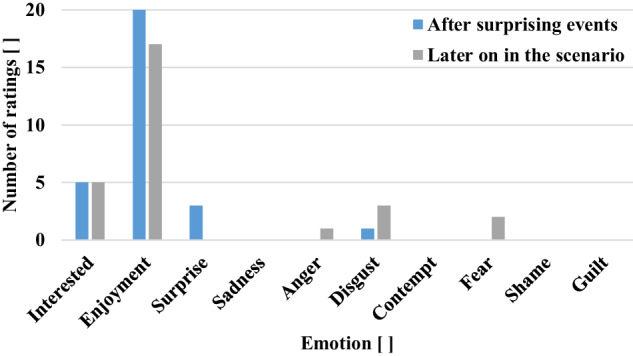
Verbal emotion rating of drivers immediately after surprising events and later on in surprise scenario.

To capture drivers by surprise, unexpected events happen right at the beginning of the respective scenario. The system then verbally asks the drivers about their feelings. The subjects are free whether or not to answer the question with any word of their own choice. Later, when the drivers’ surprise has completely worn off, they are asked again about their feelings. Here again, subjects are free not to answer or to answer with any word they choose. Fig. 13 shows the drivers’ ratings of these two verbal questions. The initial *χ*^2^ test^44^ shows no significant difference between the two ratings (*χ*^2^ = 7.23, *P* = 0.20), however replication is encouraged.

## Usage Notes

The dataset comprises about 600. To facilitate the upload and download process, the dataset has been compressed to almost 45 in the 7z format. Files with the 7z extension are compressed archive files created with 7-Zip software, which reduces the file size while preserving quality. Most common file archiving software, such as WinRAR or 7-Zip, can unpack these files. Also, in the DataExtraction.py file, the function find_and_extract_7z_files() can be used to extract the zipped files.

The scope of the dataset is provided in *.txt format, which is suitable for most text viewing programs that can process large files and analyze the data. All data files have header and time stamps. By integrating this dataset with any other dataset the used sensor for collection of the data and the sampling rate should be considered.

The broad coverage of the manD 1.0 dataset makes it suitable for various research questions. The dataset can be used to train driving behavior models using data collected from drivers during manual driving. It can be used to predict the reaction time and type of drivers in critical situations by using the data from takeover situations. Since all participants go through the same driving scenarios, the differences between individuals can be investigated. There are also some similarities between the scenarios, which provides the opportunity to compare the effects of different emotions on individuals’ driving behavior. In addition, the dataset contains data from multiple sensors that allow for the measurement of several synchronized psychophysiological factors. These measurements, along with vehicle and environmental state data, can be used to develop and train cognitive architectures and mental state models. In addition, the scenarios contain various interaction signals whose effect on the participants’ behavior can be investigated and evaluated for the development of new interaction concepts. The inclusion of data collected from participants with motion sickness experience during the experiment may be helpful for research towards this concept in the simulator. The data is mainly collected in manual driving mode and includes all sensor readings. Some examples of research questions that can be investigated using this part of dataset are the research on similarities in driving behavior of drivers with motion sickness, and the early detection of motion sickness based on physiological and behavioral data before the onset of the annoying symptoms such as headache and dizziness.

## Data Availability

The custom code “DataExtraction.py” is available^26^ from the same repository, (10.7910/DVN/SG9TMD). The code consists of functions to generate *HR* from *BVP* and *ECG* as well as functions to create images and videos of seat pressure distribution heatmaps. It also includes functions for extracting zipped files of the dataset. The code is tested and run with Python 3.11^45^.
